# Robust Individual-Cell/Object Tracking via PCANet Deep Network in Biomedicine and Computer Vision

**DOI:** 10.1155/2016/8182416

**Published:** 2016-08-25

**Authors:** Bineng Zhong, Shengnan Pan, Cheng Wang, Tian Wang, Jixiang Du, Duansheng Chen, Liujuan Cao

**Affiliations:** ^1^Department of Computer Science and Engineering, Huaqiao University, Xiamen, Fujian Province 361021, China; ^2^School of Information Science and Technology, Xiamen University, China

## Abstract

Tracking individual-cell/object over time is important in understanding drug treatment effects on cancer cells and video surveillance. A fundamental problem of individual-cell/object tracking is to simultaneously address the cell/object appearance variations caused by intrinsic and extrinsic factors. In this paper, inspired by the architecture of deep learning, we propose a robust feature learning method for constructing discriminative appearance models without large-scale pretraining. Specifically, in the initial frames, an unsupervised method is firstly used to learn the abstract feature of a target by exploiting both classic principal component analysis (PCA) algorithms with recent deep learning representation architectures. We use learned PCA eigenvectors as filters and develop a novel algorithm to represent a target by composing of a PCA-based filter bank layer, a nonlinear layer, and a patch-based pooling layer, respectively. Then, based on the feature representation, a neural network with one hidden layer is trained in a supervised mode to construct a discriminative appearance model. Finally, to alleviate the tracker drifting problem, a sample update scheme is carefully designed to keep track of the most representative and diverse samples during tracking. We test the proposed tracking method on two standard individual cell/object tracking benchmarks to show our tracker's state-of-the-art performance.

## 1. Introduction

Individual-cell/object tracking is a fundamental problem in computational biology [1–4], drug treatment effects on cancer cells [2], high-content screening [5], and computer vision [6–8]. Therefore, it has attracted much attention due to the potential value for its theoretical challenges and practical applications. Although it has been investigated in the past decades, designing a robust cell/object tracker to cope with appearance changes of a cell/object is still a great challenging task. The appearance changes of a cell/object include intrinsic (e.g., pose changes, motion blur, scale variations, and nonrigid deformation) and extrinsic (e.g., illumination variations, cluttered scenes, and occlusions) factors. Such appearance changes may make a tracker drift away from the cell/object. Moreover, because a large number of manual operations are required in existing cell tracking [9], how to design an accurate and automatic cell tracker with limited manual operations [10] is another challenge.

To capture appearance variations, most state-of-the-art trackers rely on handcrafted features to adaptively construct and update the generative or discriminative models of object appearances (e.g., principal component analysis [1, 2, 11, 12], Hough forest [13], support vector machine [14], and ensemble learning [15, 16]). By using various handcrafted features [16–26], these handcrafted feature-based tracking methods are developed for certain scenarios. Consequently, they are unable to capture the rich sematic information of a target as their generalization is not well. Therefore, they are prone to tracking failure in some challenging conditions.

Recently, deep learning [27–32] has attracted much attention in computational biology, cell biology, and computer vision. Instead of using handcrafted features, deep learning aims to automatically learn hierarchical feature representation from raw data. With the impressive performance achieved by deep learning on speech recognition [28] and image recognition [29, 31, 32], a few of early researchers [33–39] have applied it to object tracking and achieved competitive performance. However, as only the annotation of a target object in the initial frames is available, the deep learning-based trackers usually use large-scale training data to prelearn deep structure and transfer the pretrained feature representation to the tracking tasks. Consequently, the large-scale pretraining is time-consuming and the pretrained feature representation may be less discriminative for tracking a specific cell/object. Moreover, they may be sensitive to partial occlusion and pose changes due to using a single global bounding box to delineate the entire cell/object.

In this paper, we propose a robust discriminative tracking method which automatically learns feature representation without large-scale pretraining and explicitly handles partial occlusion by fusing a global structure and local details in a cell/object. Specifically, in the initial frames, an unsupervised method is firstly used to learn the abstract feature of a cell/object by exploiting both classic principal component analysis (PCA) algorithms with recent deep learning representation architectures. We use learned PCA eigenvectors as filters and develop a novel algorithm to represent a target by composing of a PCA-based filter bank layer, a nonlinear layer, and a patch-based pooling layer, respectively. Then, based on the feature learned from the above unsupervised method, a neural network with one hidden layer is trained in a supervised mode to construct a discriminative target appearance model. By exploiting the advantage of deep learning architecture, our method is able to learn a generic and hierarchical feature representation while performing more efficiently without large-scale pretraining. Compared with holistic-based models, our method simultaneously maintains holistic and local appearance information and therefore provides a compact representation of the target object. Finally, to alleviate the tracker drifting problem, a simple yet effective sample update scheme is adopted to keep track of the most representative and diverse samples while tracking. The experiments on two standard individual-cell/object tracking benchmarks (i.e., the Mitocheck cell dataset [40] and the online tracking benchmark (OTB) [41]) show that our tracker achieves a promising performance.

The rest of the paper is organized as follows.  Section 2 discusses the most related work to ours. The detailed overall framework of our tracking method is described in Section 3. The performance of our tracking method is demonstrated in Section 4. Finally, Section 5 summarizes our findings.

## 2. Related Work

Much work has been done in the area of cell/object tracking and the comprehensive review is beyond the scope of this paper. Please refer to [3, 4, 6–8] for more complete reviews on cell/object tracking and recent tracking benchmarks. In this section we briefly review some representative works on visual tracking and put our work in a proper context.

### 2.1. Individual-Cell and Object Tracking with Handcrafted Features

For decades, many tracking methods with handcrafted features have been proposed, which focus on constructing robust cell/object appearance models to handle the inevitable appearance changes of a cell/object. In [17], a mean shift-based tracking method using color histograms is proposed. Li et al. [18] propose a multiple nuclei tracking method with the intensity features for quantitative cancer cell cycle analysis. In [19], Danelljan et al. propose an adaptive color attribute-based tracking method under a coloration filtering framework. In [26], Lou et al. propose an active structured learning-based cell tracking method by combining multiple complementary features, such as position, intensity, and shape. In [12], an incremental principal component analysis-based tracking method is proposed for robust visual tracking. Recently, a variety of low-rank subspaces and sparse representations based tracking methods have been proposed [42–47] for cell/object tracking due to their robustness to occlusion and image noises. Zhong et al. [48] propose a weakly supervised learning-based tracking method, in which multiple complementary trackers are effectively fused to achieve robust tracking results. Zhou et al. [49] propose a similarity fusion-based tracking method, in which multiple features and context structure of unlabeled data are effectively utilized.

Coupled with designing handcrafted features, numerous advanced machine learning methods have been developed to further improve the tracking performances. The typical learning methods include support vector machine (SVM) classifiers [14], structured output SVM [21], online boosting [15, 20], P-N learning [50], multiple instance learning [51], and correlation filters [52–54]. In [55], for improving the tracking performance, Lou et al. incorporate a shape prior into a learning method to segment dense cell nuclei. Dzyubachyk et al. [56] utilize a level set-based method for cell tracking in time-lapse fluorescence microscopy.

Moreover, to explicitly deal with the occlusion problem, several part-based models have been proposed. In [13], Gall et al. propose a part-based voting schema via Hough forests for robust tracking. In [17], online latent structural learning is employed for a part-based object tracking method. However, the part-based tracking methods still rely on low-level features. Although tracking methods with handcrafted features usually produce more accurate results under less complex environments, they may be limited by using handcrafted features which cannot be simply adapted according to the new observed data obtained while tracking.

### 2.2. Single-Cell and Object Tracking with Deep Learning

Inspired by the success of deep learning in speech and visual recognition tasks [27–32], a few of deep learning-based tracking methods have been recently proposed [33–39] for robust cell/object tracking. In [35], based on a pretrained convolutional neural network, Fan et al. propose a tracking method for human. One of the limitations is that the pretrained convolutional neural network is fixed during the online tracking process. Wang and Yeung [34] propose an autoencoder based tracking method. Instead of using unrelated images for pretraining, Wang et al. [57] propose a tracking method which prelearns features robust to diverse motion patterns from auxiliary video sequences. However, they only evaluate the method on 10 video sequences. In [58], Li et al. effectively combine multiple convolutional neural networks for robust tracking. Within a particle filtering framework, Carneiro and Nascimento [33] use deep learning architectures to cope with the left ventricle endocardium in ultrasound data. In [36], based on the deep network of VGG, a fully convolutional neural network is proposed for robust tracking. In [37], Hong et al. propose a tracking method by learning discriminative saliency map with convolutional neural network. In [38], Ma et al. fuse the correlation filters and pretrained VGG network for robust tracking. In [39], Nam and Han propose a multidomain convolutional neural network-based tracking method.

However, these tracking methods are time-consuming due to the large-scale pretraining. Moreover, the pretrained feature representation may be less discriminative for tracking specific target objects.

## 3. The Proposed Individual-Cell and Object Tracking Algorithm

In this section, we develop our discriminative tracking algorithm via a PCANet deep network [32]. Based on a particle filtering framework, the proposed PCANet-based tracking method for individual-cell/object is schematically shown in Algorithm 1.

Specifically, the proposed tracking algorithm works as follows: the target object is manually selected in the first frame by a bounding box. Then, an unsupervised method is used to learn the abstract feature of the target object by exploiting both classic principal component analysis (PCA) algorithms with recent deep learning representation architectures. Furthermore, based on the feature learned from the above unsupervised method, a neural network with one hidden layer is trained in a supervised mode to construct a discriminative object appearance model. Meanwhile, a set of particles with associated weights is initialized within a particle filtering framework. For one incoming video frame *t*, we first predict each particle using the dynamic model. Then, we compute weights for each particle using the observation model (i.e., the discriminative appearance model). According to the obtained weights, we determine the optimal object state as the particle with the maximum weight and resample particles. Finally, the pretrained feature is updated according to the new observed data. Meanwhile, the discriminative appearance model is also incrementally updated via a simple yet effective sample update scheme which keeps track of the most representative and diverse samples while tracking. The tracking procedure continues in this iterative fashion until the end of video.

Below we give a detailed description about each component of our method.

**Algorithm 1 .**

Overview of the proposed PCANet-based tracking method for individual-cell/object is shown below.

Input is as follows:

1. Get one initialized video frame with ground-truth bounding box on a cell/object.
2. Pretrain an abstract feature of a cell/object via an unsupervised method.
3. Build a neural network-based discriminative appearance model for the cell/object based on the feature learned from the above unsupervised method.
4. Initialize a set of particles with associated weights within a particle filtering framework.

Output is as follows:

1. Predict each particle using a Gaussian function-based motion model.
2. Compute weights for each particle using a PCANet-based discriminative appearance model.
3. Determine the optimal cell/object state as the particle with the maximum weight.
4. Resample particles based on their corresponding weights.
5. Update the pretrained feature and the PCANet-based discriminative appearance model according to the newly observed data.

### 3.1. Particle Filtering

The proposed tracking algorithm is carried out using the particle filtering framework which is a Markov model with hidden state variables. Supposing that we have observations of the target object *Z*_*t*_ = [*z*_1_,…, *z*_*t*_] up to the *t*th frame, the hidden state variable *x*_*t*_ is estimated by the well-known two-step iteration (i.e., the prediction and the update steps):

(1) pxt ∣ Zt∝pzt ∣ xt∫pxt ∣ xt−1pxt−1 ∣ Zt−1dxt−1,

where *p*(*x*_*t*_∣*x*_*t*−1_) is the dynamic (motion) model between two consecutive states and *p*(*z*_*t*_∣*x*_*t*_) is the observation model which estimates the likelihood of observing *z*_*t*_ at state *x*_*t*_. The optimal object state *x*_*t*_^*∗*^ at time *t* can be determined by the maximum a posteriori estimation over *N* samples (particles) at the *t*th frame by

(2) $$\begin{array}{c} x_{t}^{*}=\overset{i=1,\ldots ,N}{\underset{x_{t}^{i}}{\arg\max}}\left\{\;p\left(\;z_{t}^{i}\;|\;x_{t}^{i}\;\right)p\left(\;x_{t}^{i}\;|\;x_{t-1}\;\right)\;\right\}, \end{array}$$

where *x*_*t*_^*i*^ is the *i*th sample of the state *x*_*t*_ and *z*_*t*_^*i*^ is the image observation predicted by *x*_*t*_^*i*^.

*Motion Estimation*. In this paper, for simplicity and computational efficiency reasons, we choose to track only the location and size. Let *x*_*t*_ = (*l*_*t*_^*x*^, *l*_*t*_^*y*^, *w*_*t*_, *h*_*t*_) denote the object state parameters including the horizontal coordinate, vertical coordinate, width, and height, respectively. We use a Gaussian distribution to model the dynamic model between two consecutive frames. 

*Likelihood Evaluation*. For each state *x*_*t*_, there is a corresponding image patch that is normalized to 32*∗*32 pixels by image scaling. The likelihood function is calculated based on the proposed discriminative appearance model; that is, *p*(*z*_*t*_∣*x*_*t*_) = *d*_*t*_, where *d*_*t*_ is an output score from the proposed discriminative appearance model.

### 3.2. The Proposed Discriminative Appearance Model from PCANet

In this section, we address the problem of how to learn a data-driven and discriminative appearance model without large-scale pretraining. In the first frame, an unsupervised method is firstly used to learn the abstract feature of a target object by exploiting both classic principal component analysis (PCA) algorithms with recent deep learning representation architectures. Then, based on the feature learned from the above unsupervised method, a neural network with one hidden layer is trained in a supervised mode to construct a discriminative target appearance model.

More specifically, we use the newly proposed PCANet deep network [32] to prelearn the abstract feature of a target object. The PCANet is a simple convolutional deep learning network composed of cascaded PCA, binary hashing, and block histograms. The work on PCANet shows that applying arbitrary nonlinearities on top of PCA projections of image patches can be surprisingly effective for image classification. Inspired by their work, we propose a PCANet-based unsupervised method to effectively learn the abstract feature of a target object and the discriminative structure between the target and background.

**Figure 1:**
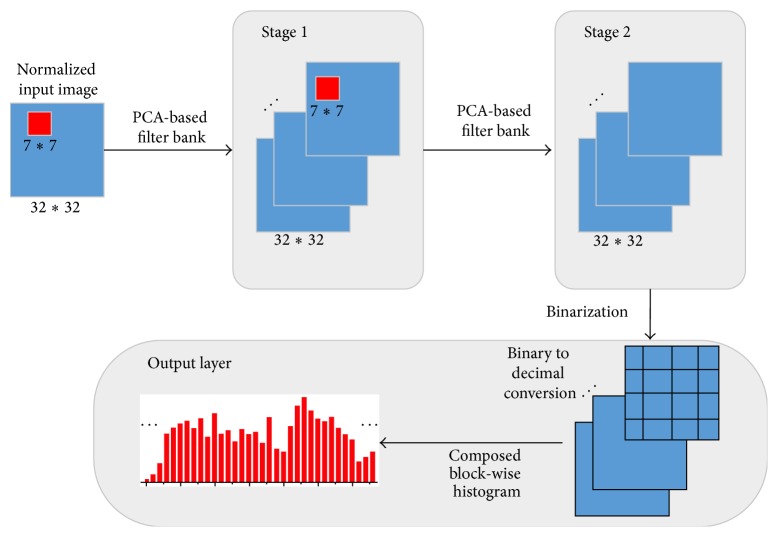
Illustration of the structure of the used PCANet deep network [32].

The PCANet model is illustrated in Figure 1, and only the PCA filters need to be learned from the training images. Following the notations of Han Chan et al. [32], we will briefly review the PCANet model.

*The Cascaded PCA*. Denote {*I*_*i*_ ∈ *ℝ* ^*m*×*n*^}_*i*=1_^*N*^ as *N* input training images and *k*_1_ × *k*_2_ as the 2D convolutional filter size. Around each pixel, PCANet takes *k*_1_ × *k*_2_ patch and collects all (overlapping) patches of the *i*th image as the training data. Then, PCANet computes projection vectors in such a way that most variations in the training data can be retained. The PCA filters in the PCANet are expressed as the leading principal eigenvectors. Similar to deep neural network, PCANet can stack multiple stages of PCA filters to extract higher level features.

*Binary Hashing and Block Histograms*. Let *L*_1_ and *L*_2_ denote the number of PCA filters in the first and second stage of PCANet, respectively. For each of the *L*_1_ input images *I*_*i*_^*l*^ for the second state, each input image has *L*_2_ real-valued outputs {*I*_*i*_^*l*^ *∗W*_*ℓ*_^2^}_*ℓ*=1_^*L*_2_^ from the second stage. These outputs are binarized via a hashing function, in which an output value is one for positive entries and zero otherwise.

Around each pixel, the vector of *L*_2_ binary bits is viewed as a decimal number. This converts *L*_2_ outputs of the *i*th input image *I*_*i*_^*l*^ back into a single integer-valued image *T*_*i*_^*l*^, where *i* = 1,…, *L*_1_. Then, each of the *L*_1_ images *T*_*i*_^*l*^ is divided into *m* overlapping or nonoverlapping blocks. PCANet compute the histogram of the decimal values in each block and concatenate all *m* histograms into one vector and denote them as *H*(*T*_*i*_^*l*^).

After this encoding process, the feature of the input image *I*_*i*_ is then defined to be the set of block-wise histograms; that is, *f*_*i*_ = [*H*(*T*_*i*_^*L*^),…, *H*(*T*_*i*_^*L*_1_^)].

**Figure 2:**
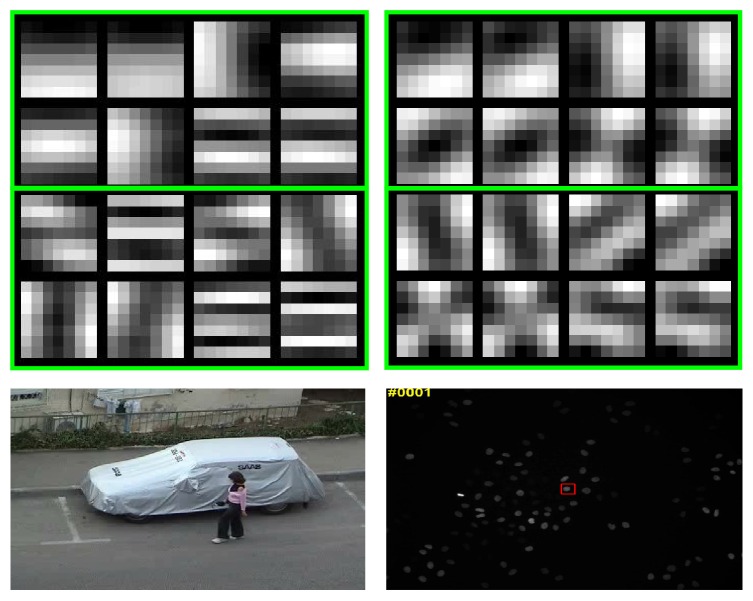
The PCA-based filters learned on the training data from the first frame of woman sequence from the online tracking benchmark (OTB) [41] and the Mitocheck cell dataset, respectively [40]. The top two rows show the eight PCA-based 7*∗*7 filters learned in first layer. The bottom two rows show the eight PCA-based 7*∗*7 filters in second layer.

To empirically illustrate the efficacy of the learned PCANet features, we check the fine-tuned filters trained on the training data from a specific tracking task. In Figure 2, we show the PCA-based filters learned on the training data from the first frame of woman sequence from the online tracking benchmark (OTB) [41] and the Mitocheck cell dataset, respectively [40]. The top two rows show the eight PCA-based 7*∗*7 filters learned in first layer. The bottom two rows show the eight PCA-based 7*∗*7 filters in second layer. It is obvious that the proposed PCANet-based model can effectively learn the useful information from the data, such as edge and corner and junction detectors.

## 4. Experiments Evaluation

This section presents our implemental details, experimental configurations, dataset, and evaluation setting. The effectiveness of our tracking algorithm (named ours-1) is then demonstrated by quantitative and qualitative analysis on the online tracking benchmark (OTB) [41] and the Mitocheck cell dataset, respectively [40]. For the sake of computational robustness, we further consider the effect of the different PCA layers in PCANet (i.e., a variety of different numbers of the PCA layers) on the tracking performance.

### 4.1. Implementation Details and Experimental Configurations

To reduce computational cost, we simply consider the object state information in 2D translation and scaling in a particle filtering framework, where the corresponding variance parameters are set to 15, 15, 0.1, and 0.1, respectively. The proposed tracking method (i.e., ours-1) is implemented in Matlab without code optimization and runs on a PC with a 2.40 GHz processor and 12 G RAM. 1,000 samples are empirically drawn for particle filtering. For each particle, there is a corresponding image region normalized to a 32*∗*32 patch. The buffer size of a temporal sliding window is set as 25. The typical training time of PCANet-based deep network is about 10 seconds in Matlab without using GPUs. Our PCANet-based tracker takes about one second to process each video frame.

### 4.2. Datasets and Evaluation Settings

#### 4.2.1. Datasets

To evaluate the performance of the proposed tracking method (i.e., ours-1) for tracking individual-cell/object, we use not only the Mitocheck cell dataset [40] but also the online tracking benchmark (OTB) [41]. The Mitocheck dataset is a time-lapse microscopic image sequence which contains higher cell density, larger intensity variability, and illumination variations. The online tracking benchmark (OTB) [41] is a collection of 50 video sequences tagged with 11 attributes which covers various challenging factors in visual tracking, such as deformation, fast motion, background clutter, and occlusion. The 50 video sequences are defined with bounding box annotations.

#### 4.2.2. Evaluation Settings

The OTB benchmark uses two different evaluation metrics: the precision plot and success plot. For the precision plot, a target object is considered to be successfully tracked on a video frame if the distance between the centers of the estimated box and the ground-truth bounding box is below a threshold. Thus, numerous precision plots can be obtained by varying the threshold values. Typically, the trackers are ranked based on the precision at threshold of 20 pixels for the precision plot. On the other hand, for the success plot, a target object is considered to be successfully located on a video frame if the predicted bounding box and the ground-truth bounding box have an intersection-over-union (IoU) overlap higher than a threshold. The success plot illustrates the percentage of frames considered to be successful. The area under curve (AUC) score is used to rank the tracking algorithms. Three different experiments are performed, that is, one-pass evaluation (OPE), temporal robustness evaluation (TRE), and spatial robustness evaluation (SRE). For TRE, the starting frame of the evaluation is randomized. For SRE, the initial bounding boxes are randomly perturbed. Please see the original paper [41] for more details. For the evaluation on the Mitocheck cell dataset [40], we just use the qualitative results to show the tracking performance due to the unavailability of ground-truth labeling.

### 4.3. Evaluation Results on the Online Tracking Benchmark (OTB)

**Figure 3:**
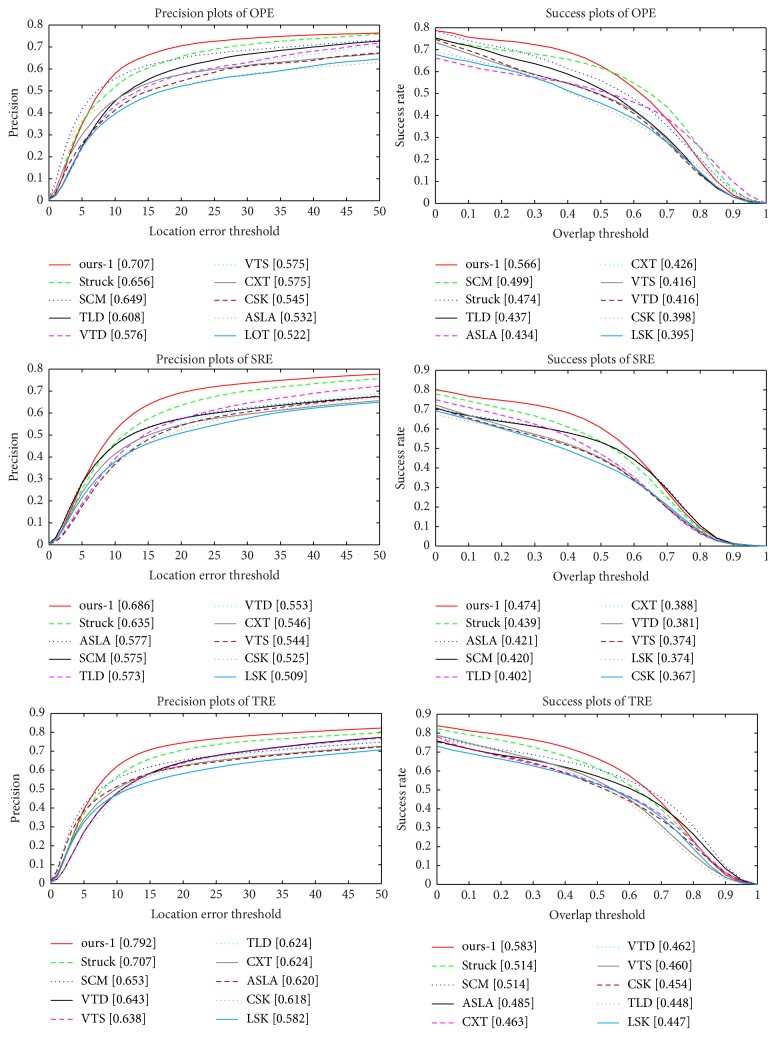
The precision and success plots of one-pass evaluation (OPE), temporal robustness evaluation (TRE), and spatial robustness evaluation (SRE) for the 50 sequences in the online tracking benchmark (OTB) [41], respectively. The legend lists the corresponding evaluation score for each tracking method. The proposed tracking method (i.e., ours-1 in red) is ranked first among the state-of-the-art trackers in both the precision and success plots.

*Overall Performance*. We quantitatively analysed the overall tracking performance, and Figure 3 shows the precision and success plots on all the 50 sequences of the top 10 tracking methods. In terms of both evaluation metrics, the proposed tracking method (i.e., ours-1) is able to obtain better results than any of the comparison methods due to the robust feature learning via online PCANet deep network. In the precision plot of OPE, the precision score of the proposed tracking method (i.e., ours-1) is 0.707, which is ranked the first place. Meanwhile, the other top four tracking methods are Struck (0.656), SCM (0.649), TLD (0.608), and VTD (0.576), respectively. In the success plot of OPE, the AUC score of the proposed tracking method (i.e., ours-1) is 0.566, which is also ranked the first place. Meanwhile, the other top four tracking methods are SCM (0.499), Struck (0.474), TLD (0.437), and ASLA (0.434), respectively. According to the precision and AUC scores, the proposed tracking method (i.e., ours-1) is comparable to the state-of-the-art tracking methods in both the precision and success plots.

**Figure 4:**
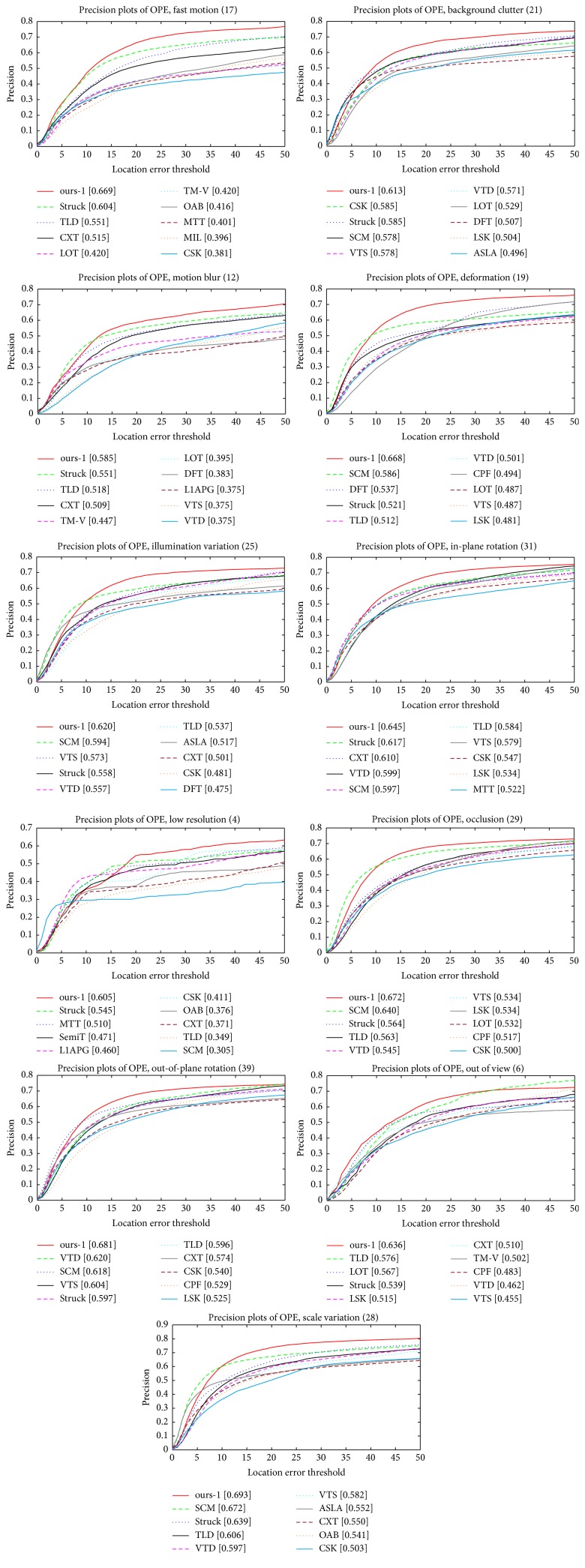
The precision plots of one-pass evaluation (OPE) for trackers on the 11 attributes. The values next to the attributes denote the number of video sequences involving the corresponding attribute.

**Figure 5:**
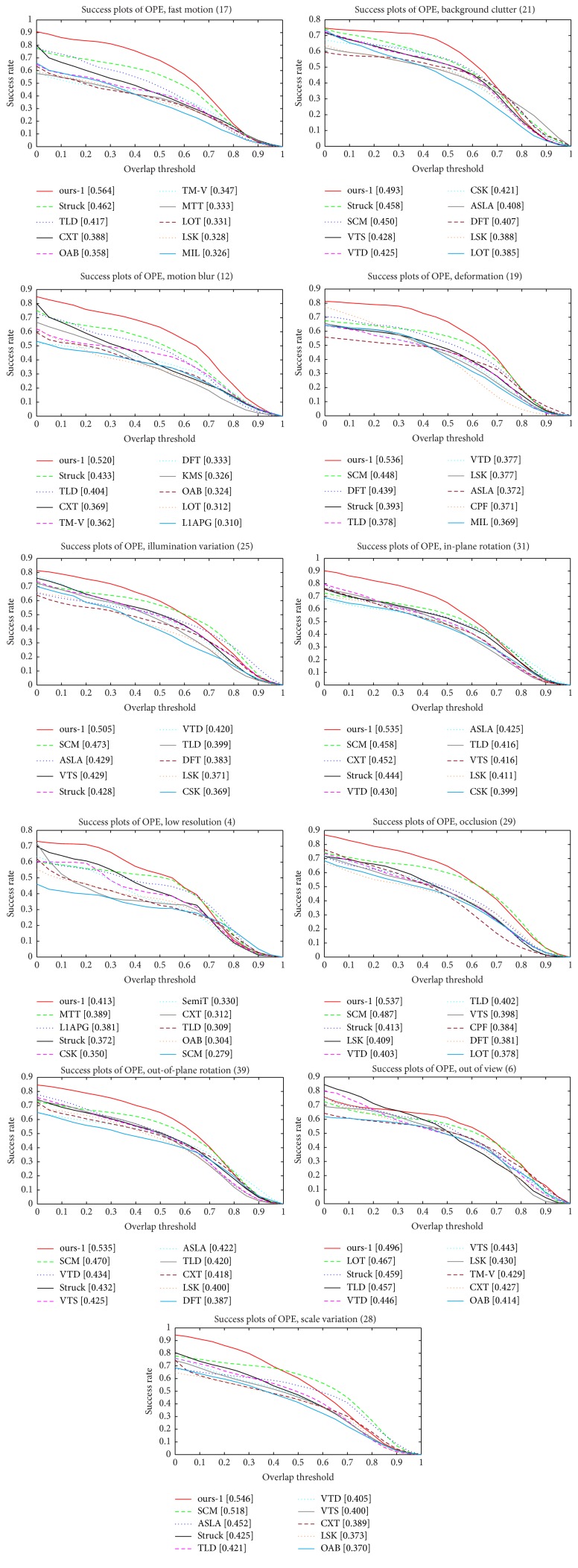
The success plots of one-pass evaluation (OPE) for trackers on the 11 attributes. The values next to the attributes denote the number of video sequences involving the corresponding attribute.

*Performance Analysis on 11 Different Attributes*. To further analyse the proposed tracking method, we validate the performance of the proposed tracker on each attribute provided in the online tracking benchmark (OTB) [41]. In the OTB, there are 11 different attributes which describe a variety of tracking challenges. Each video sequence is annotated by some attributes. We report the precision and success plots of one-pass evaluation (OPE) for trackers on the 11 attributes in Figures 4 and 5, respectively. According to Figures 4 and 5, it is easy to observe that the proposed tracking method (i.e., ours-1) provides sufficient robustness to the 11 attributes, and our tracker consistently outperforms the other trackers in most of the challenges.

**Figure 6:**
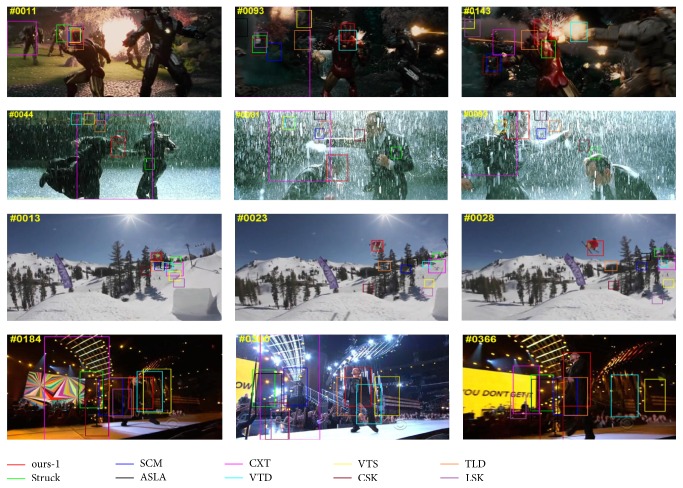
Qualitative results of the proposed tracking method (i.e., ours-1) on several challenging sequences from [41].

**Figure 7:**
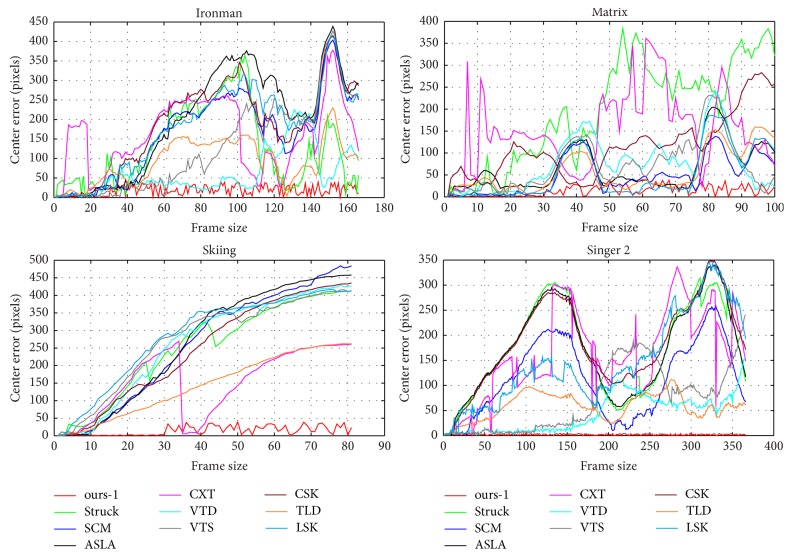
Quantitative results on the center distance error per frame for several challenging sequences from [41].

*Qualitative Results*. In Figure 6, we illustrate the qualitative results of four typical image sequences. To facilitate more detailed analysis, we further report the curves of center distance error per frame in Figure 7. As our tracker can better capture major variations in the data, we can observe that the proposed tracking method demonstrates superior performance over other tracking methods.

### 4.4. Effect of Different PCA Layers in PCANet

**Figure 8:**
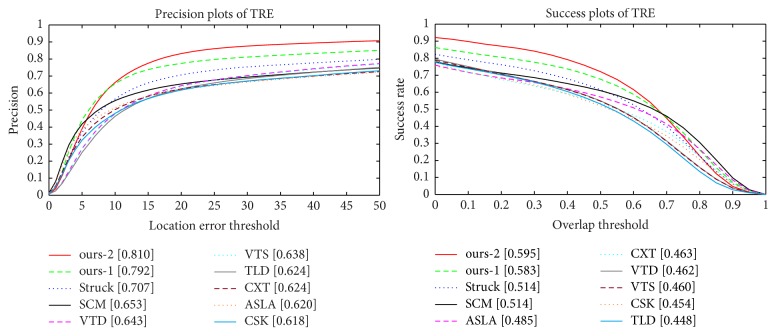
The precision and success plots of TRE for the proposed tracking method (e.g., ours-1 and ours-2) as the number of PCA filtering layers in PCANet grows. Please see the text for more details.

In this subsection, we investigate how the number of PCA layers in PCANet affects the tracking performance of the proposed method. Specifically, we compare our tracker (i.e., ours-1) with one different structure. The new variation of ours-1 is denoted as ours-2. Different to ours-1 tracker which contains two PCA filtering layers, ours-2 tracker contains three PCA filtering layers.  Figure 8 demonstrates the performance comparison of the proposed tracking method with different PCA layers in PCANet in terms of the success and precision plots of TRE on the online tracking benchmark (OTB) [41]. We observe that ours-2 tracker with three PCA filtering layers obtains a better result than that of ours-1 tracker with two PCA filtering layers. This indicates that the performance of the proposed tracking method can be further improved when the number of PCA layers in PCANet is increased. However, the improvement is not significant and is computationally inefficient.

### 4.5. Qualitative Results on the Mitocheck Cell Dataset

**Figure 9:**
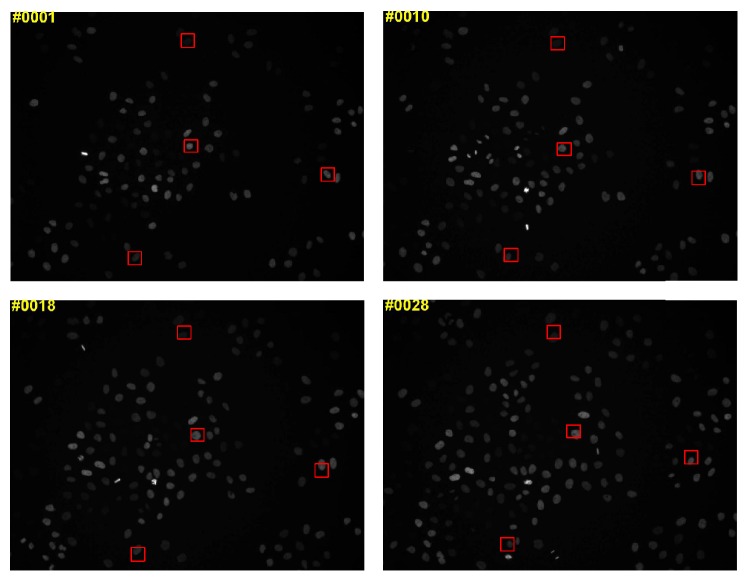
Qualitative results on individual-cell from the Mitocheck dataset [40].

To evaluate the performance of the proposed tracking method on individual-cell tracking, we test the proposed tracking method on the Mitocheck cell dataset [40]. In Figure 9, we report the qualitative tracking results of four individual-cells from the Mitocheck dataset. We can observe that the proposed tracking method simultaneously maintains holistic and local appearance information and therefore provides a compact representation of the cells. Consequently, the proposed tracking method can achieve a good performance on individual-cell tracking.

### 4.6. Discussion

In this paper, we focus on learning a robust PCANet-based appearance model for individual-cell/object tracking. According to the above experimental results on challenging dataset, the proposed tracking method has achieved promising results. However, the performance of the proposed tracker may be deteriorated when a target object is occluded over a long period of time. The reason is that the PCANet-based appearance model is updated via a simple yet concrete schema which does not explicitly detect occlusion. To address the problem, more complicated occlusion detection and forgetting schemas should be incorporated into the proposed tracker to achieve effective model updating.

## 5. Conclusion

We have proposed a robust feature learning method via PCANet deep network for robust individual-cell/object tracking in the time-lapse and 2D color imaging sequences. A cell/object is firstly effectively represented by composing of a PCA-based filter bank layer, a nonlinear layer, and a patch-based pooling layer, respectively. Then, a discriminative target appearance model is constructed by training a neural network with one hidden layer. Finally, to alleviate the tracker drifting problem, a sample update scheme is carefully designed to keep track of the most representative and diverse samples while tracking. Extensive experiments on challenging image sequences from the Mitocheck cell dataset and the online tracking benchmark (OTB) [41] validate the robustness and effectiveness of the proposed individual-cell/object tracking method.
